# A New Saponin from Tea Seed Pomace (*Camellia oleifera* Abel) and Its Protective Effect on PC12 Cells

**DOI:** 10.3390/molecules171011721

**Published:** 2012-10-01

**Authors:** Xin-Fu Zhang, Ying-Ying Han, Guan-Hu Bao, Tie-Jun Ling, Liang Zhang, Li-Ping Gao, Tao Xia

**Affiliations:** 1 Key Laboratory of Tea Biochemistry & Biotechnology, Ministry of Education and Ministry of Agriculture, Anhui Agricultural University, Hefei 230036, China; Email: zxftea@163.com (X.-F.Z.); hanyyfood@163.com (Y.-Y.H.); baoguanhu@ahau.edu.cn (G.-H.B.); lingtj@ahau.edu.cn (T.-J.L.); zhli2091@sina.com (L.Z.); 2 College of Horticulture, Qingdao Agricultural University, Qingdao 266109, China; 3 School of Life Science, Anhui Agricultural University, Hefei 230036, China; Email: gaolp62@126.com

**Keywords:** *Camellia oleifera*, tea seed pomace, triterpenoid, saponin, PC12 cell

## Abstract

A new triterpenoid saponin, oleiferasaponin A_1_, was isolated from tea seed pomace (*Camellia oleifera* Abel). The structure of oleiferasaponin A_1_ was elucidated on the basis of chemical and physicochemical evidence and was found to be 22-*O*-*cis*-2-hexenoyl-A_1_-barrigenol 3-*O*-[*β*-D-galactopyranosyl(1→2)] [*β*-D-glucopyranosyl(1→2)-*α*-L-arabinopyranosyl(1→3)]-*β*-D-glucopyranosiduronic acid. PC12 cells injured with H_2_O_2_ were used as the model to test the protective effects of oleiferasaponin A_1_. The results indicated that oleiferasaponin A_1_ can potentially prevent the H_2_O_2_-induced cell death of PC12 cells.

## 1. Introduction

The *Camellia* plant, *Camellia* (*C.*) *oleifera* Abel, has been widely cultivated as an economic or ornamental plant in many parts of China, including the Hunan, Jiangxi, Anhui, Henan, Zhejiang and Fujian provinces. The seeds of this plant are used for oil manufacture, while the byproduct, tea seed pomace, is normally discarded as waste or is used for fuel and feed after treatment in traditional Chinese industries. However, there is about 8% saponin in tea seed pomace [1]. Saponin has been commercially utilized as a foam-stabilizing and emulsifying agent [1] and is extensively used in aquaculture to eliminate unwanted fish and harmful insects in prawn ponds [2]. In addition, it is used as a medicine for the treatment of intestinal disorders [3] and burn injuries [4].

The chemical constituents of the seeds of *C amellia sinensis* var. *sinensis* have been studied extensively, and many compounds such as the theasaponins A_1_–A_3_, F_1_–F_3_ [5], A_4_, A_5_, C_1_, E_8_–E_9_, G_1_, H_1_ [6], A_6_, A_7_, B_5_ [7], E_1_, E_2_ [8] and E_3_–E_7_ [9] have been reported over the years. However, very few studies have been performed on characterizing the chemical constituents and biological activities of saponin obtained from the tea seeds of *C. oleifera*. Huang *et al*. identified a new compound, sasanquasaponin [3], which has a triterpenoid structure that is similar to the structures of some ginseng saponins [10]. Furthermore, the protective effect of this compound on endothelial cell injury has been studied. Kuo *et al*. detected the presence of camelliasaponin B_1_ in a saponin mixture that was obtained from the methanol extract of tea (*C. oleifera*) seed pomace and studied the antifungal activities of this mixture [11]. Tea saponin has also been reported to exert many pharmacological effects, including antihyperlipidemic [12], antiallergic [13], and cardioprotective effects [14]. This paper deals with the isolation and structure elucidation of a new saponin, oleiferasaponin A_1_, from tea seed pomace of *C. oleifera*, as well as its protective effect on PC12 cells.

## 2. Results and Discussion

The methanol extract of tea seed pomace (*Camellia oleifera* Abel) was dissolved in water and was separated by a nanofiltration membrane; it was then successively subjected to purification through a macroporous resin column, a silica gel column, and repeated high pressure liquid chromatography (HPLC) to yield the new compound.

**Figure 1:**
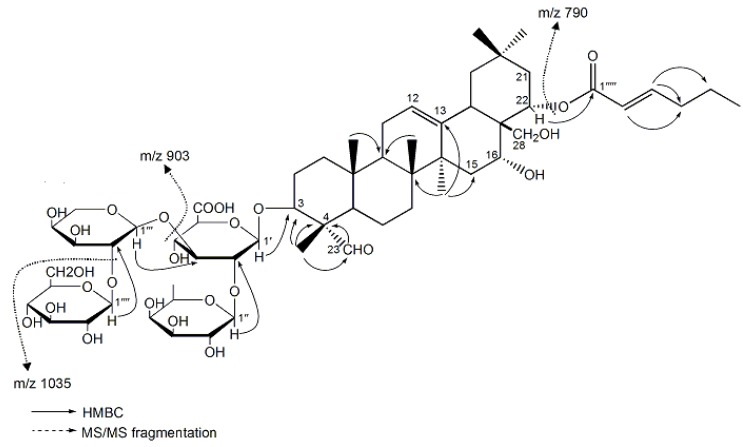
2D NMR correlations and MS/MS fragmentation of oleiferasaponin A_1_.

The new compound was visualized by spraying with 1% (w/v) Ce(SO_4_)_2_ in 10% (v/v) aqueous H_2_SO_4_, followed by heating at 120 °C, and it displayed purplish black spots on a thin-layer chromatography (TLC) plate, suggesting that it possessed the basic triterpenoid skeleton [15]. The IR spectrum showed absorption bands at 3386, 1717, 1655, 1077, and 1047 cm^−1^ due to hydroxyl; *α*, *β*-unsaturated ester; carboxy; and ether functions. The molecular formula, C_59_H_92_O_26_, was determined from the [M−H]^−^ ion at *m/z* 1215.57975 by high-resolution negative-ion electrospray ionization mass spectroscopy (ESI-MS/MS). The MS/MS fragmentation patterns (Figure 1) of the parent ion at *m/z* 1215.57975 confirmed the successive loss of a hexose (*m/z* 1035.51441 [M−H−C_6_H_11_O_6_]^−^), a pentose (*m/z* 903.47240 [M-H-C_11_H_19_O_10_]^−^), and a hexene (*m/z* 790.97736 [M−H−C_17_H_28_O_12_]^−^) unit. The ^1^H-(methanol-d_4_) and ^13^C-nuclear magnetic resonance (NMR) spectra (Table 1), which were assigned following various NMR experiments, including distortionless enhancement of polarization transfer (DEPT)-90, DEPT-135, 2D homonuclear correlation spectra (H-HCOSY), heteronuclear single quantum coherence (HSQC), and heteronuclear multiple bond correlation (HMBC) spectroscopic examination, showed signals assignable to an A_1_-barrigenol moiety. This moiety included six methyls [*δ* 0.95, 0.96, 1.07, 1.08, 1.21, 1.54 (all s, H_3_-29, 25, 26, 30, 24, 27)], a methylene [*δ* 3.72 (br s, H_2_-28)], and two methines bearing an oxygen function [*δ* 3.92 (m, H-3), 5.45 (dd, *J* = 6.0, 3.0 Hz, H-22)], an olefin [*δ* 5.39 (br s, H-12)], and four glycopyranosyl moieties—*β*-D-glucopyranosiduronic acid [*δ* 4.41 (d, *J* = 6.0 Hz, H-1′)], *β*-D-galactopyranosyl [*δ* 5.05 (d, *J* = 6.0 Hz, H-1′′)], *α*-L-arabinopyranosyl [*δ* 4.54 (d, *J* = 6.0 Hz, H-1′′′)], and *β*-D-glucopyranosyl [*δ* 4.54 (d, *J* = 6.0 Hz, H-1′′′′)], together with a *cis*-2-hexenoyl group [*δ* 0.99 (t, *J* = 8.0 Hz, H_3_-6′′′′′), 1.51 (m, H_2_-5′′′′′), 2.66 (m, H_2_-4′′′′′), 6.29 (dt, *J* = 12.0, 4.0 Hz, H-3′′′′′), 5.85 (dt, *J* = 12.0, 1.6 Hz, H-2′′′′′)]. The data were very similar to those of sasanquasaponin I [13] and camelliasaponin B_1_ [16].In addition, the signal of C-4 was markedly shifted downfield (_Δ_*δ*C = 17.2 ppm) and that of C-5 was shifted upfield (_Δ_*δ*C = −6.4 ppm), and HMBC correlations from –CHO (*δ*ppm 9.48) to C-4 showed that the 23-methyl was substituted by a –CHO moiety. Furthermore, the position of the acyl group and the structure of the oligoglycoside moiety were confirmed on the basis of HMBC correlations. Long-range correlations were observed between the following proton and carbon pairs: H-22 and C-1′′′′′, H-1′ and C-3, H-1′′ and C-2′, H-1′′′ and C-3′, and H-1′′′′ and C-2′′′. On the basis of the above mentioned evidence, the chemical structure of oleiferasaponin A_1_ was determined to be 22-*O*-*cis*-2-hexenoyl-A_1_-barrigenol 3-*O*-[*β*-D-galactopyranosyl (1→2)][*β*-D-glucopyranosyl (1→2)-*α*-L-arabinopyranosyl (1→3)]-*β*-D-gluco-pyranosiduronic acid. 

**Table 1:** ^1^H- (400MHz) and ^13^C-NMR (100MHz) data of oleiferasaponin A_1_ (in methanol-d_4_; *δ* in ppm, *J* in Hz).

No.	*δ*_C_	*δ*_H_	No.	*δ*_C_	*δ*_H_
1	39.8	2.55 (m)	3-*O*-GlcA		
2	26.1	1.53 (m)	GlcA-1′	105.3	4.41(d, 6.0)
3	86.6	3.92 (m)	GlcA-2′	78.7	3.8(m)
4	56.8		GlcA-3′	84.3	3.71(m)
5	49.1	1.37 (m)	GlcA-4′	71.1	3.86(m)
6	21.7	0.96 (m)	GlcA-5′	77.4	3.57(m)
7	33.7	1.29 (m)	GlcA-6′	172.6	
8	42.6		2′-*O*-Gal		
9	48.4	1.11 (m)	Gal-1′′	103.2	5.05(d, 6.0)
10	37.5		Gal-2′′	74.0	3.76(m)
11	25.1	1.99 (m)	Gal-3′′	75.3	3.76(m)
12	124.8	5.39 (br s)	Gal-4′′	71.4	3.55(m)
13	144.5		Gal-5′′	76.7	3.68(m)
14	41.7		Gal-6′′	62.9	3.82(m)
15	35.6	1.65(m)	3′-*O*-Ara		
16	71	4.11(br s)	Ara-1′′′	102.1	4.54(d, 6.0)
17	45.8		Ara-2′′′	83.7	3.89(m)
18	41.8	2.56 (m)	Ara-3′′′	71.5	3.69(m)
19	42.7	2.26 (m)	Ara-4′′′	67.8	4(dd, 12.0, 6.0)
20	32.5		Ara-5′′′	64.9	3.26(m)
21	43.0	2.27 (m)	2′′′-*O*-Glc		
22	73.9	5.45(dd, 6.0, 3.0)	Glc-1′′′′	108.1	4.54(d, 6.0)
23	211.2	9.48 (br s)	Glc-2′′′′	75.4	3.53(m)
24	11.3	1.21(s)	Glc-3′′′′	76.5	3.32(m)
25	17.7	0.96 (s)	Glc-4′′′′	71.6	3.64(m)
26	16.9	1.07 (s)	Glc-5′′′′	78.4	3.69(m)
27	28.2	1.54 (s)	Glc-6′′′′	63.1	3.77(m)
28	63.1	3.72 (br s)	22*-O-*(*cis*-2-Hexenoyl)		
29	34.1	0.95 (s)	1′′′′′	168.8	
30	25.7	1.08 (s)	2′′′′′	121.9	5.85(dt, 12.0, 1.6)
			3′′′′′	151.4	6.29(dt, 12.0, 4.0)
			4′′′′′	32.4	2.66(m)
			5′′′′′	23.8	1.51(m)
			6′′′′′	14.4	0.99(t, 8.0)

**Figure 2:**
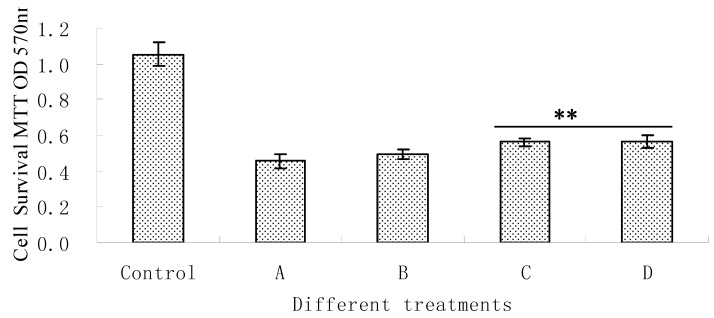
Cell protective effects of oleiferasaponin A_1_ on H_2_O_2_-induced cytotoxicity in PC12 cells (n = 8). A, 5 mM H_2_O_2_; B, 5 mM H_2_O_2_ + 5 μM oleiferasaponin A_1_; C, 5 mM H_2_O_2_ + 25 μM oleiferasaponin A_1_; D, 5 mM H_2_O_2_ + 125 μM oleiferasaponin A_1_; The values are expressed as mean ± SD. ** *p* < 0.01 with respect to the H_2_O_2_ group.

Oleiferasaponin A_1_ was tested for its protective effect on PC12 cells injured by H_2_O_2_. The cell viabilities of PC12 cells injured upon treatment with H_2_O_2_ at 5, 25, and 125 μM are shown below (Figure 2). These results show that oleiferasaponin A_1_ has potential cytoprotective activity against H_2_O_2_-induced damage.

## 3. Experimental

### 3.1. General

The following spectrometric instruments were used to obtain physical data: IR spectra were obtained on a Nicolet FTIR-8700 spectrometer (Thermo Scientific Instrument Co., Boston, USA); mass spectrometry was performed using a Thermo Scientific LTQ Orbitrap XL instrument (Thermo Electron, Bremen, Germany) equipped with an ESI source operated in the negative-ion modes; ^1^H-NMR and ^13^C-NMR spectra were obtained on a AVANCE AV 400 (400/100 MHz) spectrometer (Bruker, Fallanden, Switzerland). with tetramethylsilane as an internal standard. The following materials and equipment were used for membrane separation and chromatography: 3000-Da nanofiltration membrane, (SJM, Hefei, China), AB-8 macroporous resin column (Bonc, Cangzhou, China), ordinary-phase silica gel column (200–300 mesh; Anhui Liangchen Silicon Material Co. Ltd., Huoshan, China) and a Varian Prostar HPLC instrument (Model 325) (Varian, Mulgrave, Australia).

### 3.2. Plant Material

Tea seed pomace (*Camellia oleifera*) was collected in October 2010 from a factory in Shucheng, Anhui, China.

### 3.3. Extraction and Isolation

The tea seed pomace (20 kg) was cut and extracted three times with methanol under reflux for 3 h. Evaporation of the solvent under reduced pressure provided a brown syrup (2.2 kg). The methanol extract (1.5 kg) was dissolved in water and purified using a nanofiltration membrane. The concentrated solution (1.1 kg) was subjected to AB-8 macroporous resin column chromatography with stepwise gradients of water and ethanol (100:0, 70:30, 30:70, and 0:100, v/v) to afford four subfractions. The third subfraction (590 g) was further subjected to ordinary-phase silica gel column chromatography [CHCl_2_:CH_3_OH:H_2_O (80:60:5, v/v)] to yield six fractions. A part of fraction 5 (900 mg) was purified by HPLC [MeOH:H_2_O (30:70)] to furnish a saponin mixture (86 mg). The mixture was further purified by HPLC [acetonitrile in 0.2% AcOH:H_2_O (41:59)] to give oleiferasaponin A_1 _(23 mg).

### 3.4. Measurement of Cell Viability

PC12 cells were cultured as previously described [17]. Cells were added into the wells of a 96-well culture plate at a density of 10^5^ cells/mL for incubation before the cell viability experiments. The cells were permitted to adhere to plates for 16 h after seeding. Then, different volumes of the fresh compound stock were added to the plates to achieve ﬁnal concentrations of 5, 25, and 125 μM. After 2 h incubation with the compound, 5 mM H_2_O_2_ was added into the plates. The cells were subjected to stress for 48 h before the experimental analyses.

The antioxidant properties of oleiferasaponin A_1_ on cell viability were assessed by the 3-(4,5-dimethylthiazol-2-yl)-2,5-diphenyltetrazolium bromide (MTT) assay as previously described [18]. In brief, the MTT assay was performed by the addition of MTT solution (5 μg/mL) to each well, and 2 h later, the culture was dissolved in DMSO. The absorbance of MTT was measured using a microplate absorbance reader at 570 nm. The data are presented as the percentage versus the blank control, which represents 100% cell viability.

### 3.5. Statistical Analysis

The mean value and standard deviation in this experiment were calculated by Excel 2007 (Microcal Software Inc., Northampton, MA, USA). Data were subject to statistics analysis by using the software package SPSS Statistics 17.0 for Windows (release 17.0.1; SPSS Inc., Chicago, IL, USA, 2008). ANOVA was carried out to determine significant difference (** *p* < 0.01).

## 4. Conclusions

The new compound, 22-*O*-*cis*-2-hexenoyl-A_1_-barrigenol 3-*O*-[*β*-D-galactopyranosyl(1→2)][*β*-D-glucopyranosyl(1→2)-*α*-L-arabinopyranosyl (1→3)]-*β*-D-glucopyranosiduronic acid, was isolated from tea seed pomace (*Camellia oleifera* Abel) and identified as a triterpenoid saponin on the basis of spectral analysis and chemical evidence. Oleiferasaponin A_1_ is a compound that can potentially prevent the H_2_O_2_-induced death of PC12 cells.
